# Precision exercise in older adults with early Alzheimer’s disease: The study protocol of the FIT-AD Sequential, Multiple Assignment, Randomized Trial (SMART)

**DOI:** 10.1186/s13063-025-09040-0

**Published:** 2025-10-15

**Authors:** Fang Yu, Michael Todd, Dereck Salisbury, Molly Maxfield, Jeremy Pruzin, Rodney P. Joseph, Yi Su, Danni Li, Elsa Baena, David Coon

**Affiliations:** 1 Arizona State University, Edson College of Nursing and Health Innovation, Phoenix, USA; 2 University of Minnesota, Minneapolis, USA; 3 Banner Alzheimer’s Institute, Phoenix, USA; 4 Olive View, UCLA Medical Center, Los Angeles, USA

**Keywords:** Exercise, Alzheimer’s disease, Precision medicine, Biomarker

## Abstract

**Background:**

Aerobic exercise is promising for preventing Alzheimer’s disease (AD) and AD-related dementia (ADRD), but exercise trials have shown mixed results. An important, understudied factor potentially contributing to mixed results is individual differences in aerobic fitness responses to moderate-intensity continuous training (MICT). This trial will test the effects and mechanisms of 6 months of aerobic exercise tailored on aerobic fitness response to MICT in community-dwelling older adults with early symptomatic AD. We aim to (I) test the effects of aerobic exercise on aerobic fitness, white matter hyperintensity volume (WMHv), and patient-centered outcomes in older adults with early symptomatic AD; (II) determine the best exercise to improve aerobic fitness and reduce non-responses over 6 months; and (III) examine the mechanisms of aerobic exercise’s action on cognition.

**Methods:**

This stage II trial is a Sequential, Multiple Assignment, Randomized Trial (SMART). The hypothesis is that MICT augmented with high-intensity interval training (HIIT) or combined aerobic and resistance exercise (CARE) will improve aerobic fitness, WMHv, and AD/ADRD plasma biomarkers. This trial will enroll 108 dyads (participants and their study partners). Participants (*n* = 108) are randomized using a 2:1 allocation ratio to 3-month MICT or 6-month stretching control. After the initial 3-month intervention period for participants assigned to MICT, aerobic fitness is measured with peak oxygen consumption (VO_2peak_) from a cycle-ergometer exercise testing and the shuttle walk test to identify non-response (< 5% increase). Non-responders are subsequently re-randomized (1:1) to either HIIT or CARE for 3 months. Responders continue MICT. All participants are followed for an additional 6 months post-intervention. Primary outcomes are VO_2peak_ measured at 0, 3, 6, 9, and 12 months and WMHv at 0, 6, and 12 months. Secondary outcomes include memory, physical function, behavioral and psychological symptoms of dementia (BPSD), quality of life (QoL), caregiver burden, and AD plasma biomarkers. This trial has 80% power, assuming 18% and 25% attrition at 6 and 12 months, respectively, to detect changes in aerobic fitness.

**Discussion:**

Individual differences in VO_2peak_ responses were reported in older adults with AD/ADRD previously but how this affects response to exercise interventions is unknown, Precision exercise tailored to VO_2peak_ is critical to advance exercise research in AD.

**Trial registration:**

ClinicalTrials.gov NCT05877196. Registered on May 25, 2023.

## Administrative information

Note: the numbers in curly brackets in this protocol refer to SPIRIT checklist item numbers. The order of the items has been modified to group similar items (see http://www.equator-network.org/reporting-guidelines/spirit-2013-statement-defining-standard-protocol-items-for-clinical-trials/).

**Table:** 

Title [1]	Precision exercise in older adults with early Alzheimer’s disease: The study protocol of the FIT-AD SMART
Trial registration {2a and 2b}	Name of the registry: ClinicalTrials.gov Trial registration number: NCT05877196 Date of registration: 5/25/2023 URL of trial registry record: https://clinicaltrials.gov/study/NCT05877196?cond=Alzheimer%20Disease&intr=MICT&rank=1&tab=history
Protocol version {3}	November 4, 2024, version 11
Funding [2]	This research is funded by the National Institute on Aging of the National Institutes of Health R01AG076566-01A1.
Author details {5a}	Fang Yu^1^, Michael Todd^1^, Dereck Salisbury^2^, Molly Maxfield^1^, Jeremy Pruzin^3^, Rodney P. Joseph^1^, Yi Su^3^, Danni Li^2^, Elsa Baena^4^, David Coon^1^ ^1^Arizona State University Edson College of Nursing and Health Innovation ^2^University of Minnesota ^3^Banner Alzheimer’s Institute ^4^Olive View-UCLA Medical Center
Name and contact information for the trial sponsor {5b}	Kristina McLinden, Kristina.Mclinden@nih.gov
Role of sponsor {5c}	The content is solely the responsibility of the authors and does not necessarily represent the official views of the National Institutes of Health.

## Introduction

### Background and rationale {6a}

Alzheimer’s disease (AD) cannot be cured and affects 6.2 million Americans, with estimates indicating it will affect 14 million Americans and cost > $1.1 trillion by 2050 [1]. Aerobic exercise is a promising intervention for preventing and treating AD but has shown mixed effects on clinical outcomes such as cognition [2], which is likely caused by an important, understudied factor—individual differences in aerobic fitness responses. Individual differences in fitness response to habitual moderate-intensity continuous training (MICT), long established in young adults [3–5], are more prominent in older adults [6]. Although trials in older adults with AD and related dementias (ADRD) showed a dose-response relationship between aerobic exercise and cognition [7–9], inter-individual differences in aerobic fitness response to MICT was first reported by the FIT-AD Trial [10]. Hence, precision exercise that focuses on individual differences [11–13] is needed with adaptive exercise such as high-intensity interval training (HIIT) and combined aerobic and resistance exercise (CARE) which have been shown to mitigate aerobic fitness non-responses among older adults without AD [14]. Moreover, animal studies [15] strongly support aerobic exercise modifying AD’s AT(N) biomarkers: Amyloid-beta (Aβ), Tau, and Neurodegeneration [16] though human studies are limited with conflicting findings [17–20].

### Objectives {7}

The purpose of this manuscript is to describe the study protocol for FIT-AD SMART, which aims to test the effects and mechanisms of 6-month aerobic exercise that is tailored on aerobic fitness response to MICT in community-dwelling older adults with early symptomatic AD. FIT-AD refers to Function Improvement through aerobic exercise Training in older adults with AD and SMART refers to Sequential, Multiple Assignment, Randomized Trial design. The FIT-AD SMART was built on findings from the FIT-AD Trial that showed individual differences in aerobic fitness responses to MICT to:

- Aim I. Test the effects of aerobic exercise on aerobic fitness, white matter hyperintensity (WMHv), and patient-centered outcomes in older adults with early symptomatic AD. H1a: Aerobic exercise (MICT, MICT + HIIT, MICT + CARE) will increase aerobic fitness and reduce WMHv over 6 and 12 months more than stretching. H1b: Aerobic exercise will improve patient-centered outcomes over 12 months in comparison to stretching.
- Aim II. Determine the best exercise to improve aerobic fitness and reduce aerobic fitness non-responses over 6 months in older adults with early symptomatic AD. H2a: MICT + HIIT improves aerobic fitness the most, followed by MICT + CARE, then MICT only, and then stretching. H2b: HIIT reduces the number of MICT non-responders more than CARE.
- Aim III. Examine the mechanisms of aerobic exercise’s action on memory in early symptomatic AD. H3: Changes in plasma Aβ_42/40_, phospho-tau181 (ptau181), total tau (t-tau), and neurofilament light chain (NfL) mediate memory responses to exercise.

### Trial design {8}

**Fig. 1:**
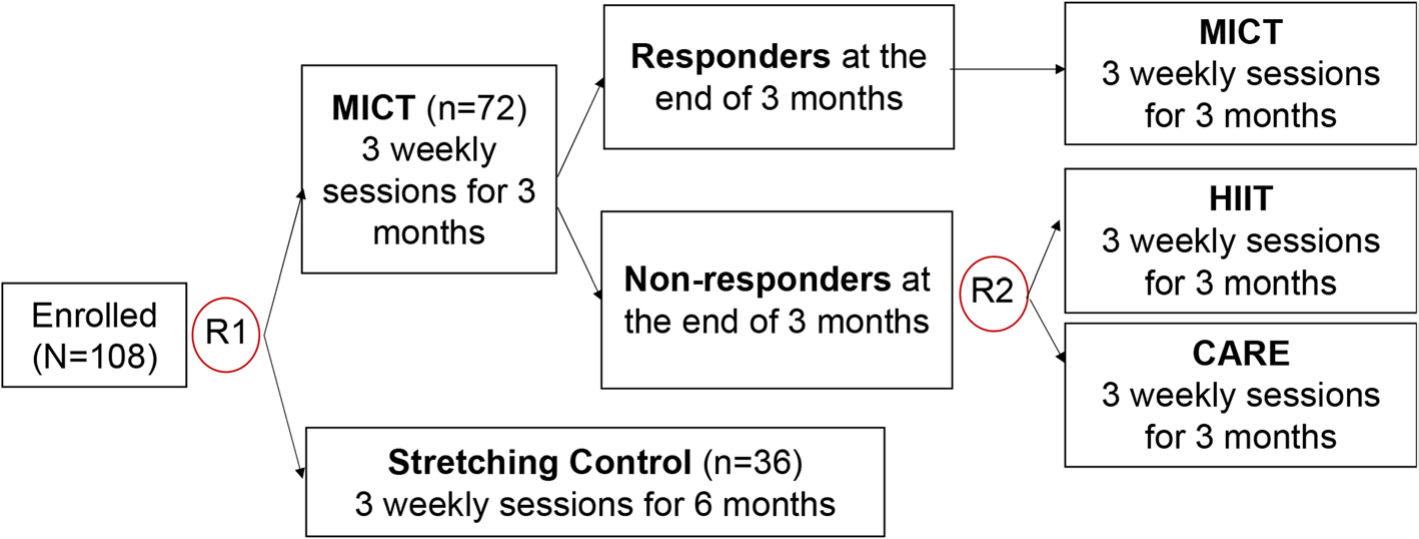
The FIT-AD SMART design

FIT-AD SMART is a single-site Stage II (according to the NIH Stage Model) sequential, multiple assessment, randomized trial. One hundred and eight (*n* = 108) of community-dwelling older adults with early symptomatic AD are randomized using a 2:1 allocation ratio (randomization 1) to 3-month MICT or 6-month stretching control, after baseline data collection. Their primary family caregivers are also enrolled as study partners. VO_2peak_ is re-assessed at the end of 3 months and is used to identify non-responders (defined in the Methods section) in participants assigned to the MICT group. Non-responders are subsequently re-randomized on a 1:1 allocation ratio to either HIIT or CARE for 3 months (randomization 2). Responders continue MICT for another 3 months (Fig. 1). Both randomizations are stratified by baseline age (66–75, 76–85, 85 + years) using random permuted blocks of 3 and 6. Study outcomes are assessed at 0, 3, 6, 9, and 12 months by trained data collectors who are blinded to group allocation.

## Methods: participants, interventions, and outcomes

### Study setting {9}

The FIT-AD SMART is a single-site trial. Screening, exercise testing, blood collection and storage, and non-MRI data collections occur on the Downtown Campus of Arizona State University (ASU), located in Phoenix, AZ. Exercise sessions are delivered in local gyms such as YMCAs or in senior communities. MRI is obtained at Banner Alzheimer’s Institute and other Banner facilities when a larger physical size of the MRI machine is needed.

### Eligibility criteria {10}

Early symptomatic AD is defined as mild cognitive impairment (MCI) and mild AD dementia using the 2011 criteria [21] through reviews of medical records (lab, notes, cognitive testing), screening cognitive test, health history, and MRI. The criteria for MCI include [1] 21 ≤ Montreal Cognitive Assessment (MoCA) score ≤ 26 (education-corrected), [2] Functional Activities Questionnaire (FAQ) < 9, [1] absence of dementia, [2] cognitive impairment not due to other causes, including major neurologic, psychiatric, and substance abuse disorders, e.g., Parkinson’s disease, head trauma, cortical stroke, major depression, and alcohol abuse, and [3] lack of MRI evidence indicating normal pressure hydrocephalus, brain tumor, subdural hematoma, significant post-traumatic encephalomalacia, and one or more large cortical infarction. The criteria for mild AD dementia include (1) insidious onset and clear-cut history of worsening cognition that interferes with ability to function at work or at usual activities by self/family report or observation, (2) decline from previous levels of functioning by self/family report or observation, (3) 11 ≤ MoCA score < 21, (4) FAQ ≥ 9, (5) cognitive impairment not due to other causes, including major neurologic, psychiatric, and substance abuse disorders, e.g., Parkinson’s disease, non-AD dementia, head trauma, cortical stroke, major depression, and alcohol abuse, and (6) lack of MRI evidence indicating normal pressure hydrocephalus, brain tumor, subdural hematoma, significant post-traumatic encephalomalacia, and one or more large cortical infarction. The eligibility criteria for both participants and study partners are detailed in Table 1.

**Table 1:** Eligibility criteria

Inclusion criteria	Exclusion criteria
**Older adults with early symptomatic Alzheimer’s disease (AD)**	
o Early symptomatic AD (mild cognitive impairment or mild AD dementia [probable or possible]) o Community-dwelling o ≥ 65 years old o Verified exercise safety o Having a qualified study partner o Volunteering to donate 18 mL blood 5 times o Verified MRI safety	o Resting heart rate ≤ 50 or ≥ 100 bpm due to arrhythmia after 5-min quiet resting o American College of Sports Medicine exercise contraindications, e.g., acute myocardial infarctions o New or unevaluated signs, symptoms, or diseases o Abnormal findings during symptom-limited cycle-ergometer test o Enrollment in another intervention aimed at improving cognition o Moderate to strenuous exercise > 150 min a week in the previous 6 months o Geriatric depression scale-15 ≥ 9, on ≥ 2 anti-depression medications, or poorly managed or unstable depression o Generalized Anxiety Disorder-7 ≥ 10
**Inclusion criteria for study partners**	
o 18 years old or older o Contact with the participant ≥ 2 times per week for ≥ 6 months o Know the participant’s memory status and ability to perform daily activities such as managing money, driving, shopping, and preparing meals o Consent to participate	

### Who will take informed consent? {26a}

During in-person interview, the study staff trained to conduct consent will assess decisional capacity of participants and obtain informed consent from participants and their study partners.

### Additional consent provisions for collection and use of participant data and biological specimens {26b}

Efforts will be made to limit the use and disclosure of participants’ personal information, including research studies and medical records, to people who need to review this information. All collected data such as demographic, physical, and mental data, brain scan, and blood specimens will be de-identified. De-identified data will be stored on a secure database and blood specimens in − 80° freezer at ASU. These de-identified data may be shared with other researchers to answer new research questions. In this sense, complete secrecy cannot be promised. Organizations such as the Institutional Review Board (IRB) that may inspect participant data and biological specimens.

## Interventions

### Explanation for the choice of comparators {6b}

The comparator is stretching that is matched to cycling duration, but at light intensity (≤ 30% of heart rate [HR] reserve [HRR] or rating of perceived exertion [RPE] ≤ 9). HRR is determined based on the peak cycle-ergometer exercise test during screening as the difference between peak and resting HRs. This comparator group was selected to equalize staff-participant interactions without affecting aerobic fitness.

### Intervention description {11a}

The initial aerobic exercise intervention is MICT on recumbent stationary cycles. The dose of MICT is prescribed as 50–75% of HRR and 9–14 RPE and starts at 50–60% of HRR or RPE 9–11 for 30 min. Cycling intensity and duration is alternatively increased by 5% of HRR (or 1-point on the RPE) or 5-min increments as tolerated up to 65%–75% of HRR (or RPE 12–14) for 50 min a session over time.

### Criteria for discontinuing or modifying allocated interventions {11b}

After 3 months of MICT, aerobic fitness non-response is determined in a three-hierarchical order as (1) < 5% increase in VO_2peak_ if peak cycle-ergometer exercise test with indirect calorimetry is completed at sufficient effort (e.g., respiratory exchange ratio ≥ 1.0 or HR ≥ 85% of the age-predicted max HR). (2) If exercise test is refused (e.g., participant wishes not to wear testing mask), non-response is indicated by < 10% increase in peak aerobic power (peak watts/kg) on the exercise test. (3) For participants who do not complete the exercise test, non-response is indicated by < 36.5-m increase in the distance of shuttle walk test (SWT). Non-responders are re-randomized 1:1 to HIIT or CARE for 3 months, stratefied by baseline age and random permuted blocks of 3 and 6. Responders continue MICT for 3 more months.

HIIT. An evidence-based “Scandinavian-style” HIIT protocol is used [22, 23]. This protocol includes 40 min of HITT comprising 5 sets of 4-min high-intensity cycling followed by 4-min moderate-intensity cycling. After a 10-min warm-up, participants progress to moderate intensity before the first set of HIIT. High intensity is prescribed as 80–85% HRR or RPE 15–16 for the first 8 weeks and increased to 80–90% HRR or RPE 15–17 after week 8. Following the HITT session, a 10-min cool-down period is conducted.

CARE. CARE includes 20-min resistance exercise followed by 30-min cycling. Resistance exercises target six major muscle groups (chest press, shoulder press, squats, rows, leg press, calf raises), are performed on exercise machines, and are adapted for any mobility or musculoskeletal issues reported by participants. Intensity is based on estimated one-repetition maximal strength (1RM) and progress following guidelines for older adults [24]. It starts with 2 sets of 10–12 repetitions at moderate intensity (50–60% 1RM, 9–11 RPE) and progressively increases to 70–85% 1RM or 13–16 RPE by week 7. Rest intervals are 1–2 min between sets. Cycling intensity is 65–75% HRR or RPE 12–14, like exercise prescription used during the MICT.

### Strategies to improve adherence to interventions {11c}

All sessions are standardized and matched for duration, using objective HRR and subjective Borg RPE measures of intensity. The exercise physiologist prescribes exercise intensity for each participant based on screening exercise testing. Exercise sessions include up to 3 participants and are supervised by an interventionist. The interventionist assesses participant’s HRs with HR monitor, activity tracking, pulse oximeter, or manual method and measures blood pressure to guide participants for 5-min warm-up, prescribed exercise, and 5-min cool-down (or 10-min cool-down for HIIT). The interventionist assesses and documents participant HRs, RPE, talk test, and over-exertion signs/symptoms every 5 min and blood pressure every 10–15 min. Participants can leave after HR and blood pressure return to pre-exercise levels. The proper use of the RPE is continuously reinforced.

Attendance and session dose achieved are monitored in weekly intervention meetings. Any issues are addressed, and staff re-training are conducted. Based on lessons from our previous trials, we (1) make up any missed regular sessions each week, e.g., the participant completed 2 sessions in a week, and efforts will be made to make up the 3rd session for the week; (2) start to offer the 4th session (makeup session) per week if attendance is < 100%; (3) check each session report weekly and implement strategies to improve adherence to session intensity/duration; and (4) make up for absent weeks.

### Relevant concomitant care permitted or prohibited during the trial {11d}

Concurrent enrollment in another intervention trial is prohibited during the trial.

### Provisions for post-trial care {30}

No provision for ancillary and post-trial care and for compensation to those who suffer harm from trial participation.

### Outcomes {12}

Primary outcomes are aerobic fitness measured at 0, 3, 6, 9, and 12 months and WMHv measured at 0, 6, and 12 months. Secondary outcomes (memory, physical function, BPSD, QoL, caregiver burden) and plasma biomarkers (Aβ_42/40_, p-tau181, t-tau, NfL) are assessed at 0, 3, 6, 9, and 12 months. Exploratory outcomes include discrete cognitive domains (attention, working memory, processing speed, executive function, visuospatial ability, language, other MRI and plasma biomarkers, and study partner outcomes).

#### Primary outcomes

Aerobic fitness is assessed with the peak oxygen consumption (VO_2peak_) from the cycle-ergometer test [24] (primary test) and the maximal walking distance in meters from the SWT [25] (secondary test). The cycle-ergometer test is performed in a post-absorptive state. Participants begin to cycle at a comfortable speed, with the intensity increased every minute by increasing the resistance equivalent to 15–20 W, to reach volitional fatigue or termination criteria per American College of Sports Medicine guidelines: inability to maintain cadence, exhaustion, desire to stop, and clinical indications in 8–12 min [24]. VO_2_, HR, and heart rhythm are continuously monitored via indirect calorimetry and electrocardiogram, and RPE in the last minute of each stage and at peak. VO_2peak_ is defined as the average VO_2_ measured in the final 8 breaths immediately prior to the termination of the test. The SWT stresses participants to peak performance by walking circuits around 2 markers 9 m apart while being paced by a recording. Each minute, pacing increases to promote volitional fatigue. The SWT lasts 5–15 min with a test–retest reliability of 0.76–0.99 [25]. Distance walked on the SWT is used as a measure of aerobic fitness.

WMHv is assessed from MRI FLAIR images using the lesion segmentation toolbox [26]. MRI acquisition: A comprehensive MRI sequence protocol is performed on a Discovery MR 750 3 T MRI system and a 32-channel head coil using ADNI-compatible sequences optimized for our scanner [27, 28]. The MRI protocol includes a sagittal 3D T1-weighted volumetric IR-FSPGR pulse sequence to assess brain structure; a sagittal 3D T2-weighted FLAIR pulse sequence for WMHv and cerebrovascular disease; a T2* heme-weighted GRE sequence for microhemorrhages; an axial 3D pCASL pulse sequence to assess brain perfusion; a diffusion tensor imaging (DTI) pulse sequence for white matter integrity; a field map pulse sequence to correct functional MRI and DTI images for spatial distortions; an echo planer functional MRI sequence as participants rest quietly with their eyes open for resting state brain networks; and an oblique high-resolution volumetric pulse sequence for hippocampal subfields. MRI data are archived using an XNAT system and standard QC/QA procedures are utilized. MRI analysis: MRI will be analyzed using FreeSurfer (v7.1) [29] and semi-automated segmentation algorithms [30].

#### Secondary outcomes

Memory is measured with the AD Uniform Data Set Wechsler Memory Scale-Revised Logical Memory Story A and B Immediate and Delayed Recalls and Craft Story 21 immediate and delayed recall [31]. Physical function is assessed by the FAQ, Short Physical Performance Battery (SPPB) [32], Alzheimer’s Disease Cooperative Study—Activities of Daily Living (ADCS-ADL) [33], Mini Balance Evaluation Systems Test [34], dynamometer, and 6-min walk test (6MWT) [35, 36]. SPPB tests balance, gait speed, and sit to stand (score 0–12, > 0.90 reliability) [21]. ACDS-ADL evaluates the ability to perform ADLs with Cronbach’s alpha 0.64–0.87 and intraclass correlation coefficients 0.62–0.73 [33]. Mini Balance Evaluation Systems Test is a clinical balance assessment tool that shows excellent criterion validity across populations with neurological conditions (*r* = 0.72–0.85) [34]. Muscle strength is measured with Biodex muscle function test (lower extremity strength) and grip strength (upper extremity) dynamometer. 6MWT records peak walking distance in 6 min (0.98-0.99 reliability) [37, 38]. BPSD is measured with the Neuropsychiatric Inventory Questionnaire-Caregiver (NPI-Q) for presence/severity of 12 BPSD in the previous month (score 0–36, 0.91 interscale correlation) [37]. Burden is assessed using the 4-item Zarit Burden Interview (score 0–110 [0.83–0.89 internal consistency] [38]) and NPI-Q. NPI-Q evaluates caregiver distress due to BPSD (score 0–60, 0.92 interscale correlation) [37]. QoL is tested with the 13-item QoL-AD (score 13–52, Cronbach’s *α* 0.84–0.88 [39]).

Plasma biomarkers include Aβ_42/40_, p-tau181, t-tau, and NfL. Standard collection and processing protocols [40–43] are followed to reduce pre-analytical variations that could affect biomarker levels: (i) collect blood samples after at least 8 h of fasting (only water, black coffee, black tea, and medications allowed) and at the same time in the morning (between 8:30 am and 11 am) and (ii) collect blood at least 24 h after the last intervention session. The phlebotomist follows a venous-blood collection protocol and collect a total of 18 mL of blood into 3 6-mL plasma (EDTA-treated) tubes, then store the samples on wet ice until processing. The Clinical Research Services lab technician processes and aliquots the specimens using the established protocol and stores aliquoted samples in a − 80 °C freezer. We will perform biomarker measurements in year 5 to be cost-effective and reduce variability in measurements using the best assays available then and use a rigorous process to authenticate these key biochemical resources.

#### Exploratory variables and covariates

Discrete cognitive domains are assessed with the number span test forward for attention [44], number span test backward for working memory [44], Trail Making Test (TMT) Part A for processing speed [45], TMT Part B for executive function [45], Benson complex figure for visuospatial ability [46], multi-lingual naming test [47], phonemic test F and L (P and M for Spanish speakers), and animals/vegetables list generation for language [48]. MRI and non-AT(N) plasma markers, study partner outcomes, and potential covariates are assessed as detailed in Table 2.

**Table 2:** Variables, measures, and data collection timepoints (S: screening; 0: baseline)

Variables	Measures (categorical data; otherwise, continuous data)	S	Month				
			**0**	**3**	**6**	**9**	**12**
**Primary outcomes**							
Aerobic fitness	Peak cycle-ergometer test; shuttle walk test	x		x	x	x	x
WMHv	Magnetic resonance imaging (MRI)	x			x		x
**Secondary outcomes**							
Memory	AD Uniform Data Set Wechsler Memory Scale-Revised Logical Memory Story A and B, Craft Story 21 Recall		x	x	x	x	x
Physical function	Functional Activities Questions*, 6-min walk test, Short Physical Performance Battery, ACDS-ADL**, Mini Balance Evaluation Systems Test, Biodex muscle function test, grip strength		x	x	x	x	x
BPSD	Neuropsychiatric Inventory-Caregiver (NPI-Q)*		x	x	x	x	x
Quality of life	Quality of Life-AD**		x	x	x	x	x
Burden	4-item Zarit Burden Interview***, NPI-Q***		x	x	x	x	x
**Plasma biomarkers**							
Amyloid-beta (Aβ)	Plasma Aβ_42/40_ ratio		x	x	x	x	x
Tau	Plasma phosphorylated tau 181		x	x	x	x	x
Neurodegeneration	Plasma total tau, neurofilament light chain		x	x	x	x	x
**Potential covariates**							
Demographics	Age, education, sexº, race/ethnicityº, family historyº, living arrangementº, occupationº, retirement, family historyº	x					
Severity of cognitive impairment	Montreal Cognitive Assessment	x		x	x	x	x
Depression	Geriatric Depression Scale-15	x		x	x	x	x
Anxiety	Generalized Anxiety Disorder-7	x		x	x	x	x
APOE genotype	Allele combinationsº		x				
Vascular risk	Framingham Vascular Risk Score		x				
Lifestyle	Leisure Time Satisfaction, Physical Activity Scale for the Elderly		x	x	x	x	x
Sleep efficiency	Actigraphy**, Consensus Sleep Diary**		Monthly				
Comorbidity	Charlson Comorbidity Index	x		x	x	x	x
Medications	Numbers of drugs and drug classes	x		x	x	x	x
Condition changes	Medical conditions, medications, events (e.g., falls), health service use		x	x	x	x	x
Fall risk	Timed Up and Go Test, Mini-Best Test, Fall Calendar, Falls Follow-up		x	x	x	x	x
Adherence	Attendance rate, % sessions meeting prescription		x	x	x		
Social interaction	Engagement and Independence in Dementia Questionnaire**		x	x	x	x	x
**Exploratory variables**							
Executive function	Trail Making Test Parts A and B, number span test forward and backward		x	x	x	x	x
Visuospatial ability	Benson complex figure		x	x	x	x	x
Language	Multilingual naming test, phonemic test F and L (P and M for Spanish speakers), animal and vegetables list generation		x	x	x	x	x
Other biomarkers	MRI hippocampal volume, cortical thickness, whole-brain atrophy; emerging blood biomarkers		x		x		x
Study partner outcomes	Dyadic Relationship Scale***, Positive Aspects of Caregiving***, Personal Well-Being Index***, CES-D-10***, Leisure Time Satisfaction Scale***		x	x	x	x	x

Note. Aerobic fitness and WMH volume assessed during screening will be used as 0 month

^*^Completed by study partners on participants

^**^Completed by both participants and study partners on participants

^***^Completed by study partners on selves

measures are completed by participants unless noted with *, **, ***

measures are continuous data unless noted with o that refers to categorical data

### Participant timeline {13}

**Fig. 2:**
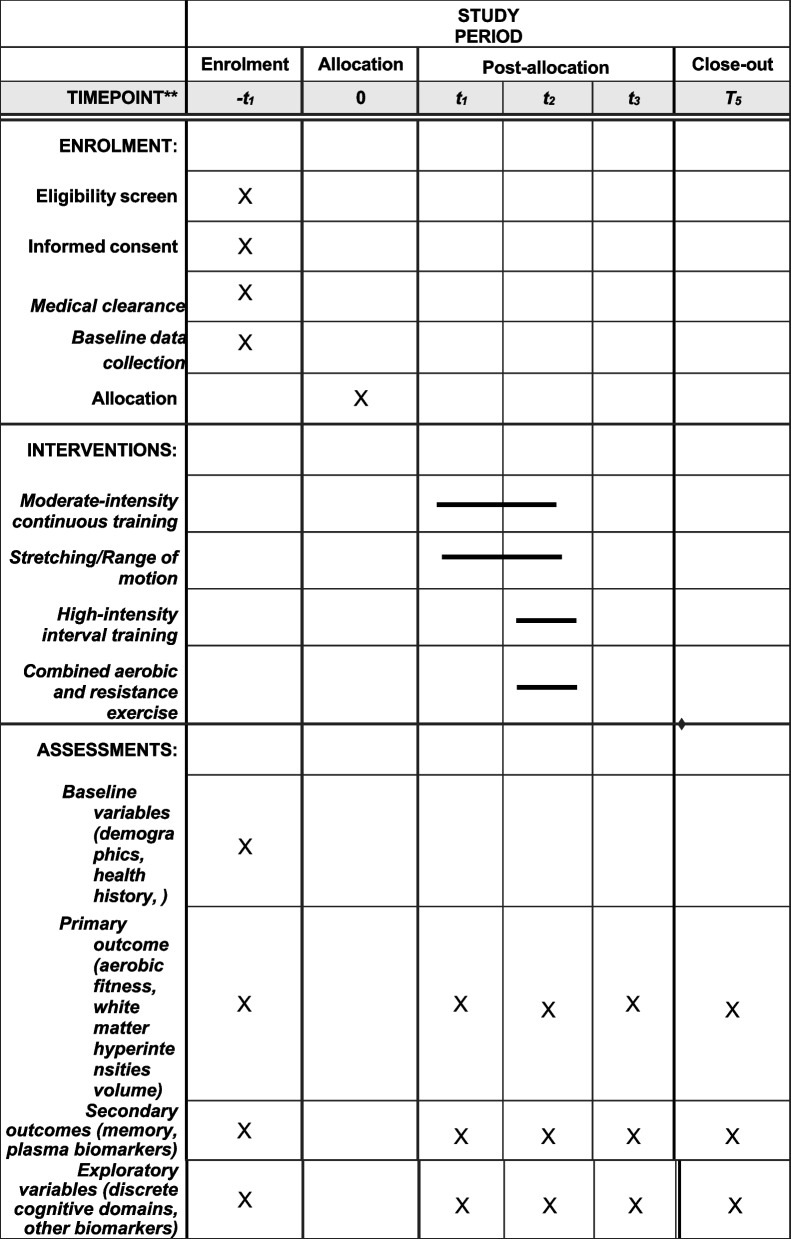
Schedule of enrollment, interventions, and assessments

Participants are recruited through referrals, registries, community events, social media, and newspaper advertisement. Respondents are screened over the phone, in-person, medical clearance, and exercise testing/MRI. The phone screen assesses cognitive symptoms, exercise and MRI contraindications. During in-person interview, informed consent or surrogate consent/assent is obtained, and global cognition is assessed with MoCA. After the in-person interview, medical clearance is obtained from participant’s primary care providers and cardiologist for those with significant cardiac history. Once medical clearance is received, participants undergo the symptom-limited cycle-ergometer exercise test to further rule out unknown heart conditions and prescribe individualized exercise doses. If no abnormality is identified from the exercise test, participants will undergo MRI. Those without MRI abnormality will proceed to baseline data collection of outcomes (Table 2). If any clinically significant abnormality was identified, participants will be referred to their providers for further evaluation and can only re-engage with the trial with medical re-clearance. Afterwards, they will be formally enrolled in the trial and randomized by the study staff designated to perform randomization. Participants will engage in their assigned exercise for 6 months and be followed for another 6 months. Outcomes (Table 2) will be assessed at 3, 6, 9, and 12 months (Fig. 2).

### Sample size {14}

Assuming 18% attrition at 6 months and 25% attrition at 12 months, the sample size of 108 participants should result in a population of *n* = 89 at 6 months and *n* = 81 at 12 months. Under H1a, tests comparing participants assigned to MICT vs. control at R1, 89 complete cases (60 intervention [MICT, MICT + HIIT, MICT + CARE]; 29 control) at 6 months should result in 80% power to detect significant between-group differences in VO_2peak_ and WMHv changes, assuming a standardized between-group difference of *d* = 0.65 (based on a clinically meaningful difference in VO_2peak_ of 3.12 mL/kg/min) and SD = 4.8 (derived from previous research [49]), a pre-post correlation (*r*) in VO_2peak_ of 0.50, and *α* = 0.05 [50]; at 12 months, 81 complete cases (54 intervention; 27 control) should result in 80% power to detect differences of *d* ≥ 0.60, assuming pre-post *r* of 0.50 and *α* = 0.05. For models under H1b, 89 complete cases at 6 months should result in 80% power to detect between-group differences in change of *d* ≥ 0.57 for memory, quality of life (QoL), physical function, and behavioral and psychological symptoms of dementia (BPSD) scores, assuming pre-post *r* of 0.50 and *α* = 0.05; at 6 and 12 months, 81 complete cases should afford power to detect differences in change of *d* ≥ 0.60. For H2a, simulations conducted in R with the latest version at the time of data analyses (20,000 replications for each of 7 effect size scenarios) indicate that tests for a monotonic ordering of group means (i.e., MICT + HIIT > MICT + CARE > MICT > stretching) on VO_2peak_ should have power ≥ 80% for orderings comprising small MICT + HIIT vs. MICT + CARE and MICT + HIIT vs. MICT differences (*d*s = 0.25–0.35) and moderate MICT only vs. stretching differences (*d*s = 0.40–0.55). For H2b, assuming *n* = 13 per group, logistic-regression models are 80% powered to detect large between-group (MICT + HIIT vs. MICT + CARE) differences in number of responders (vs. non-responders), e.g., ≤ 2 non-responders in MICT + HIIT vs. ≥ 11 non-responders in MICT + CARE at *α* = 0.05. For H3, potential mediation of exercise effects (**X**: MICT [*n* = 59] vs. control [*n* = 30]) on memory (**Y**s) through AD/ADRD plasma biomarkers (**M**s) is ≥ 80% powered to detect mediated effects comprising modest direct associations (*r*^2^s ≥ 0.12) between groups (at R1) and each putative mediator (X → M link) and between each mediator and memory (M → Y link) [51].

### Recruitment {15}

Participants are recruited through a variety of strategies such as referrals, registries, community events, study flyer distributions, support groups, presentations, exhibits, social media, and newspaper advertisement. We also recruit a general population by leveraging our extensive network with organizations and expand new partnerships through a confianza triangle, where research teams and our community partners, such as promotores (community health workers). We forge connections to participants by establishing trust with organizations that the community also trusts, thus strengthening our referral stream.

## Assignment of interventions: allocation

### Sequence generation {16a}

Prior to recruitment, the statistician created 2 randomization schedules using random permuted blocks of 3 and 6 participants in the study’s Research Electronic Data Capture (REDCap) system.

### Concealment mechanism {16b}

The REDCap randomization modules are only accessible to designated staff such as the study coordinator and interventionist to reveal group assignment.

### Implementation {16c}

The study statistician generated the allocation sequence. The study coordinator and project manager enroll participants. The study coordinator or her designated interventionist assigns participants to interventions.

## Assignment of interventions: blinding

### Who will be blinded {17a}

All investigators (except for the statistician) and outcome data collectors are blinded after assignment to interventions. They do not have access to REDCap randomization modules. Group allocations are concealed to the investigators during trial implementation. Once participants are enrolled, the data collectors only interact with participants for data collections.

### Procedure for unblinding if needed {17b}

The study interventionists and the participants are not blinded. Unblinding to investigators (except for the statistician) and data collectors is not permissible during the trial.

## Data collection and management

### Plans for assessment and collection of outcomes {18a}

All staff are required to complete the onboarding, semi-annual and booster training until they are 100% compliant with the study protocol. Intervention delivery is entered into the REDCap database in real time. Outcomes are collected with paper and pencil and are double checked for completeness, accuracy, and data entry.

### Plans to promote participant retention and complete follow-up {18b}

We proactively identify potential dropouts and missed visits to address them promptly. If a participant passes away, we will follow up with study partners regarding the circumstance of death. If a participant intends to withdraw, we will respect the participant’s choice. All attempts will be made to collect data to allow for inclusion in the analysis and reasons for withdrawal will be recorded. If staff non-compliance is found, particularly when non-compliance results in retention or follow-up issues, the study coordinator will notify the PI immediately and staff re-training will be performed within a week to avoid repeating the problem. Any missing data will be assigned to a data collector for collection. We will track and analyze dropouts, missed visits, and loss to follow-up for the overall sample and across groups and consider them as potential covariates in analyses.

### Data management {19}

The PI is responsible for the integrity and validity of the study data. The PI and the study coordinator oversee all participant records, adherence to the protocol by all staff, screening and recruitment logs, percent of exercise sessions attended, missed sessions, compliance, retention, adverse events, and study outcomes, and ensure data creation, completeness, quality check, and audits. The PI and statistician develop and implement a data audit and edit plan to ensure data accuracy and completeness.

We collect data primarily electronically using a de-identified REDCap study database via a secure web interface. Data collected on paper will be manually entered into the REDCap database. Data collected on paper will be double checked for accuracy, completeness, and data entry accuracy by the staff and research assistants (RAs) who collected data and by another staff or RA. The web server of REDCap database is housed on secure servers operated by Arizona State University and are backed up regularly. Access to the system by username and password requires specific permission from the PI.

### Confidentiality {27}

All paper and electronic forms will be identified with a unique ID number only to protect participants’ and study partners’ privacy. Paper copies of data will be stored in a dedicated office space and kept in locked file cabinets with limited access. The linkage between the participant’s name and the screening ID will be kept separately and accessible by study staff involved in the screening process and blinded to all investigators. The study coordinator and interventionists will have access to HIPAA data to ensure participant safety and care coordination in case of adverse events (AEs). Screening IDs are first assigned during screening. Study IDs are assigned to those enrolled in the study. The link between participants’ names and screening/study IDs will be accessible only to the study staff participating in the screening process to allow the transfer of screening information into the study database.

### Plans for collection, laboratory evaluation, and storage of biological specimens for genetic or molecular analysis in this trial/future use {33}

Plasma biomarker specimens are stored on wet ice until the lab technician of Clinical Research Services processes and aliquots them. Aliquoted samples are stored in a − 80 °C freezer. The Clinical Research Services staff handle the collection, processing, and storage of blood specimen. Specimen are transported accordance with the institutional standards of practice. The specimens will be stored beyond their use to satisfy the objectives of the trial and be available to other researchers after the trial via a data sharing agreement.

## Statistical methods

### Statistical methods for primary and secondary outcomes {20a}

In the preliminary analyses, univariate (e.g., means, SDs, frequencies) and bivariate (e.g., ANOVA/Kruskal–Wallis, chi-square, correlation) statistics and plots will be used first to examine distributions of and associations among study variables; to identify potential multivariate outliers, influential cases, and implausible values; to explore missing-data patterns; and to check for systematic between-group differences at baseline. To further investigate if model assumptions are met, preliminary linear-regression models predicting 6-month outcomes from group assignment will be estimated; results will inform the choice of link function (e.g., natural log), error distribution (e.g., negative binomial), and/or data transformation. The internal consistency of each multi-item composite measure will be evaluated. Analyses and plotting will be conducted in R 4.1, SAS 9.4 (Aims I–II), and Mplus 8.7 (Aim III). Holm’s approach [52] and fixed-sequence testing procedures [52–54] will be used to control for an overall false discovery rate across multiple tests.

To address H1a, we will estimate between-group differences in aerobic fitness or WMHv change from baseline to 6 months and baseline to 12 months using an ANCOVA-type approach, a generalized linear model (GLM) in which the randomized group (e.g., MICT vs. control) predicts aerobic fitness or WMH volume at 6 months (or 12 months), adjusting for baseline aerobic fitness or WMHv and potentially relevant covariates and confounders identified in preliminary analyses. To address H1b, we will estimate GLMs paralleling those used to address H1a, but with memory, QoL, physical function, BPSD, and caregiver burden as outcomes. We will also perform similar analyses for exploratory outcomes (Table 2).

To address H2a, we will estimate contrasts corresponding to a monotonic ordering of the means [55] in which mean aerobic fitness at 6 months is the highest in the MICT + HIIT group, followed by MICT + CARE, then MICT only, and then stretching. Unlike a typical linear trend analysis, this approach does not require that the conditions (groups) represent equally spaced intervals along a quantitative continuum (e.g., min/day aerobic exercise).

To address H2b, we will estimate a multivariable logistic regression predicting 6-month responses (non-responder vs. responder) from the R2 group assignment (MICT + HIIT vs. MICT + CARE), adjusting for 3-month VO_2peak_.

To address H3, we will first estimate direct effects of condition initially randomized to group (**X**: a dichotomous indicator of MICT vs. control) on each 6- and 12-month outcome (**Y**: memory). Next, we will estimate indirect (mediated) effects of group (**X**) on memory (**Y**) via one or more 6- or 12-month measure(s) of potential mediators (**M**: e.g., plasma Aβ_42/40_) with structural equation path models using approaches described and implemented by Imai et al. [56], VanderWeele [57], and Muthén et al. [58, 59]. Where warranted, we will adjust estimates of indirect effects for covariates and for X × M interaction effects on outcomes. We will conduct sensitivity analyses to evaluate assumptions about these potential confounders [56]. We will also estimate magnitudes of indirect effects [60].

All analyses will follow the intention-to-treat (ITT) approach to provide unbiased tests of treatment effects. The ITT population will be defined as all randomized participants. We will first examine associations between baseline covariates and missingness in outcomes. Along with dropout records, findings will inform model development to generate multiple complete datasets and, where warranted, the construction of analytic models applied to these datasets, under the assumption that data are missing at random. Imputed datasets will be analyzed separately, and the results pooled to yield estimates of model parameters and associated standard errors. Diagnostic analyses and plots will be used to assess the quality of the imputations [61] and the possibility that data may be missing not at random. Sensitivity analyses based on pattern mixture models with varying assumptions regarding effects of potential missing data on outcome values (e.g., shifts in means) will be used to examine robustness of findings based on missing-at-random assumptions.

### Interim analyses {21b}

There are no interim analyses and stopping guidelines.

### Methods for additional analyses (e.g., subgroup analyses) {20b}

We collect three sets of biological variables: demographics (age, sex, race/ethnicity); *APOE* genotype; and MRI/blood biomarkers. MRI/blood biomarkers are study outcomes. We will treat demographics and *APOE* genotype as covariates in our main analyses and will conduct exploratory analyses to examine how these variables moderate treatment effects.

### Methods in analysis to handle protocol non-adherence and any statistical methods to handle missing data {20c}

Protocol non-adherence will be controlled for during data analysis. The ITT population will be defined as all randomized participants. We will first examine associations between baseline covariates (e.g., group, demographics, baseline outcome values) and missingness in outcomes. Along with detailed study records of reasons for dropout, these findings will inform model development to generate multiple complete datasets and, where warranted, the construction of analytic models applied to these datasets, under the assumption that data are missing at random. Imputed datasets will be analyzed separately, and the results pooled to yield estimates of model parameters and associated standard errors. Diagnostic analyses and plots will be used to assess the quality of the imputations [61] and the possibility that data may be missing not at random. Sensitivity analyses based on pattern mixture models with varying assumptions regarding effects of potential missing data on outcome values (e.g., shifts in means) will be used to examine robustness of findings based on missing-at-random assumptions.

### Plans to give access to the full protocol, participant-level data, and statistical code {31c}

We will make the full protocol, participant-level data, statistical code, and associated documentation available upon publications of the main findings.

## Oversight and monitoring

### Composition of the coordinating center and trial steering committee {5d}

Not applicable because this trial is a single-site study.

### Composition of the data monitoring committee, its role and reporting structure {21a}

The Data Safety and Monitoring Board (DSMB) is comprised of three external scientists in geriatric medicine, exercise, and biostatistical/clinical trials who were appointed by the National Institute on Aging (NIA). One DSMB member was assigned the role of Chair and Safety Officer and the contact person for the DSMB. During the study period, the DSMB members have no direct involvement with the study. The chairperson is responsible for overseeing the semi-annual meetings, developing the agenda in consultation with the NIA program officer and the PI, and communicating the recommendations of the DSMB to the NIA program officer who conveys to the PI.

### Adverse event reporting and harms {22}

All adverse events will be documented with an adverse event or safety form, discussed with the PI and any pertinent co-investigator(s). Any safety concerns deemed greater than a mild severity is immediately reported to DSMB and IRB for review and guidance. Depending on how study staff learn of the safety concern, information is gathered on the report form via phone, electronic, or in-person interaction.

### Frequency and plans for auditing trial conduct {23}

Audits of entered data will be conducted regularly. The intervals for monitoring data and participant safety depend on the type of data: (1) outcome data are audited as they are collected and outlined in the previous paragraph; (2) adherence to the protocol are monitored weekly; (3) the study coordinator monitors all staff, screening, recruitment logs, compliance, retention, and adverse events weekly; and (4) the DSMB meet semi-annually. In addition, we perform audits of all aspects of the trial when turnover of key staff such as study coordinator occurs.

### Plans for communicating important protocol amendments to relevant parties (e.g., trial participants, ethical committees) {25}

Important protocol modifications, e.g., changes to eligibility criteria, outcomes, and analyses, will be communicated to the DSMB (including program officers from the NIA) via emails and meetings for approval after obtaining IRB approval. Changes that affect consent content will be communicated to participants by study staff through in-person meetings or over the phone.

### Dissemination plans {31a}

Data and results from this trial will be disseminated via conference presentations, manuscript publications, and data sharing. We will implement a data sharing plan in year 5. We will work with our statisticians and staff to clean the data and create a database with de-identified data and bio-samples for data sharing. All data sharing requests will be reviewed and approved by the Data Sharing Steering Committee composed of selected investigators. We will develop a Data Sharing Request Form and a Data Use Agreement Form for internal and external researchers to submit their requests.

## Discussion

Aerobic exercise is a promising non-pharmacologic AD treatment but has shown mixed effects on cognition and other outcomes such as physical function, likely due to an important, understudied factor: individual differences in aerobic fitness responses. The gold-standard measure of aerobic fitness is VO_2peak_ [24]. Individual differences in VO_2peak_ responses, long established in adults [3–5], were more prominent in older adults [6] and first reported in those with AD dementia in the FIT-AD Trial [10]. Other AD/ADRD trials report large variance in VO_2peak_ changes from MICT as well [7–9].

Mechanistically, animal studies strongly support aerobic exercise modifying AD’s AT(N) biomarkers [15, 62–75]. Similar human studies are limited. Aerobic exercise showed mixed effects on neurodegeneration [17–20], but reduced brain WMHv accumulation [30]. One study employed plasma Aβ_42_ to examine aerobic exercise effects in MCI [76]. Overall, AT(N) biomarkers are seldom studied in exercise trials [77].

Precision exercise focuses on individual differences in intervention responses [11–13] and is ideally suited to fill these research gaps to optimize effects of aerobic exercise in AD. Precision exercise can identify MICT non-responders early to initiate alternative interventions such as HIIT or CARE. HIIT [78–80] and CARE [81–87] have shown greater effects on VO_2peak_ than MICT in older adults. Lastly, 3-month MICT is adequate to improve VO_2peak_ [88, 89]. Our FIT-AD Trial using 6-month MICT showed that VO_2peak_ peaked at 3 months and was stable from 3 to 6 months [90–92], making identification of MICT non-responders at 3 months in ADRD an appropriate decision point to initiate HIIT or CARE. Improving aerobic fitness responses can help determine how, when, and to whom to adjust aerobic exercise interventions to potentially maximize the effects of aerobic exercise on cognitive outcomes in AD.

### Trial status

This protocol represents IRB protocol version 11 dated on November 4, 2024. Recruitment was formally launched on June 22, 2023, and is anticipated to complete on March 31, 2027.

## Data Availability

Data and materials will be available to users under a data sharing agreement as suggested by the NIH that provides for (1) a commitment to using the data only for research purposes and not to identify any individual participant; (2) a commitment to securing the data using appropriate computer technology; (3) a commitment to destroying or returning the data after analyses are completed; (4) all data sharing requests must be reviewed and approved by the PI; and (5) non-duplicative request to previous requests. Requesters will submit the study data request form and the data sharing agreement. The investigator team will review and approve the request (PI, exercise co-investigator, and biostatistician).

## Abbreviations

Aβ: Amyloid beta
AD: Alzheimer’s disease
ADCS-ADL: Alzheimer’s Disease Cooperative Study—Activities of Daily Living
ADRD: AD and related dementias
ASU: Arizona State University
BPSD: Behavioral and psychological symptoms of dementia
CARE: Combined aerobic and resistance exercise
DSMB: Data and Safety Monitoring Board
DTI: Diffusion tensor imaging
HIIT: High-intensity interval training
HR: Heart rate
HRR: HR reserve
IRB: Institutional Review Board
ITT: Intention-to-treat
MCI: Mild cognitive impairment
MICT: Moderate-intensity continuous training
MoCA: Montreal Cognitive Assessment
MRI: Magnetic resonance imaging
NfL: Neurofilament light chain
NPI-Q: Neuropsychiatric Inventory Questionnaire-Caregiver
p-tau181: Hyperphosphorylated tau 181
QoL: Quality of life
REDCap: Research Electronic Data Capture
RPE: Rating of perceived exertion
TMT: Trail Making Test
VO_2peak_: Peak oxygen consumption
WMHv: White matter hyperintensity
1RM: 1-Repetition maximum
6MWT: 6-Minute walk test
